# Mobile healthcare platforms' sustainability: The perspective of health information quality

**DOI:** 10.3389/fpubh.2022.1059252

**Published:** 2023-01-06

**Authors:** Xiaoyang Sun, Wenjing Zhou, Ying Feng

**Affiliations:** ^1^School of Finance and Economics, Jiangsu University, Zhenjiang, China; ^2^School of Management, Jiangsu University, Zhenjiang, China

**Keywords:** sustainability, health information quality, evolutionary game theory, reputation mechanism, mobile healthcare platforms

## Abstract

As an emerging form of medical organization, Chinese mobile healthcare (mHealth) platforms are inherently linked to the continuous use of users, which depends on the quality of the health information provided. However, improving the health information quality of mHealth platforms is still a problem that needs to be studied and solved in order to make the platforms sustainable. Based on the reputation mechanism, this study creates a behavioral evolutionary game model for health information providers (physicians) and managers (mHealth platforms), explores the evolution process and evolutionarily stable strategy of the behaviors in various situations, and uses numerical simulation technology to analyze mHealth platforms' constraints and the influencing factors of health information quality. This study presents three key findings. First, considering reputation, health information managers and mHealth platform providers should not unilaterally optimize health information. Instead, mHealth platforms should have active quality control, and physician groups should provide high-quality health information, which is the ideal evolution of the model. Second, the rewards that physicians receive from patients and mHealth platforms for providing quality health information, the reputation benefits, the penalties that physicians suffer for providing low-quality health information, and the increased probability and cost of rent-seeking behavior that physicians may choose can effectively promote the choice of physicians to provide high-quality health information. Third, the reputation gain of mHealth platforms, the probability of mHealth platforms being exposed, and increased losses suffered from exposure can effectively promote the choice of mHealth platforms to control the quality of health information. This study can provide a theoretical basis for mHealth platforms' health information quality control, which is conducive to the healthy and sustainable development of mHealth platforms and the improvement of user satisfaction.

## 1. Introduction

In the context of an increasingly aging population, the overall shortage and inefficient allocation of medical resources is an important issue, and people's awareness of healthcare has grown. This has led to the emergence of a large number of mobile healthcare (mHealth) platforms, developed in conjunction with 5G networks and smartphones. Currently, there are many concepts of mHealth. According to the World Health Organization (WHO), mHealth is defined as “the use of mobile wireless technologies for health” (1). According to a WHO's study, mHealth provides medical services and information *via* mobile communication technology. Specifically, in the mobile Internet field, it is a doctor–patient platform based on medical and health applications (apps) in mobile terminal systems, such as Ping An Good Doctor and Chunyu Doctor, in China. These platforms provide a new way for patients to access health information and for physicians to provide health information to the public (2). Its use can enhance access to healthcare and improve the health status of individuals (3). According to Media Research, in 2020, the number of China's mHealth users reached 661 million, and the size of China's mHealth market reached 54.47 billion yuan. mHealth has advantages that traditional healthcare cannot match, being able to overcome space, time, and even organizational barriers (4–6). It offers patients convenient, economical, and personalized access to medical services (such as appointment registration, online consultation, self-diagnosis, etc.), as well as health information (such as popular science articles, popular science videos, classic cases, etc.), which has great potential to improve the health of chronic disease patients (7–9). mHealth platforms are increasingly being seen as an innovative solution to the medical difficulties of 1.3 billion Chinese people, bringing tremendous value to hospital systems, patients, and government supervision, and potentially contributing to the development of the medical sector in the rest of the world (10).

However, mHealth platforms, as an emerging form of medical organizations, have also encountered many problems in the process of their rapid development. mHealth platforms are not well-structured, doctors' and patients' information is not symmetrical, and the participation rate of doctors is low (11). There is a general concern about the quality of health information available on mHealth platforms (12). A large amount of distorted health information can easily mislead the subjective perception of users, thus failing to meet their needs. For example, mHealth platforms can recklessly exaggerate certain health products, present so-called famous doctors, and pose a threat to patients' privacy. Online health information sources are scattered, carriers are diverse, and standards are lacking, which presents users with great challenges regarding inquiries regarding, and the selection of, health information, potentially even posing a threat to their lives. The sustainable and healthy development of mHealth platforms requires users' continuous use, which in turn requires these platforms to meet the needs of users, while the quality of health information on the platforms directly affects the continuous use of users. The quality of health information on these platforms is influenced by the behavior of the providers (physicians) and managers (mHealth platforms) of health information. The sustainability of mHealth platforms depends on the dynamic balance between the interests of health information providers and managers.

Therefore, how to optimize and improve the quality of health information on mHealth platforms so as to increase user satisfaction, increase user stickiness, achieve the dynamic balance of health information providers and managers, and ensure the sustainable development of mHealth platforms has become a major issue in mHealth services. The present study considers the reputation mechanism and constructs a game model for the behavioral evolution of health information providers (physicians) and managers (mHealth platforms). The main contributions of this study are as follows: First, it innovatively studies the sustainable development of mHealth platforms from the perspective of health information quality. Second, as opposed to previous studies that have analyzed the quality of health information from the perspective of the content, this study examines quality from the perspective of health information providers and managers. Third, this study introduces the reputation mechanism to reveal the impact of reputation on the quality of health information on mHealth platforms.

The remainder of the study is divided into the following sections: Section 2 reviews the literature; Section 3 describes the evolution of the game model construction and details the stability analysis; Section 4 discusses the simulation results; Section 5 describes this study's contributions and limitations.

## 2. Literature review

### 2.1. Mobile healthcare platforms

The rapid growth of mHealth platforms has entailed increasing research interest, with the debate regarding its advantages and disadvantages seemingly never-ending (13), while also being considered an important tool to promote the future of healthcare services (14). Using online health services provided by mobile platforms can improve users' health literacy and health self-management (15–17). The application of mHealth platforms can achieve the rational allocation of medical resources and improve the quality of medical services (18), thus realizing the rapid flow of medical information and the sharing of medical resources. The use of mHealth platforms by doctors and patients is a novel and effective method for tracking and managing chronic diseases (19). College students use mHealth platforms to find health-related information, including how to lose or control their weight (20) and to monitor their diet (21). Using online healthcare platforms, healthcare providers can provide many forms of professional support (22), especially in terms of professional health information (23). The focus of mHealth platform research has shifted to the platform-based medical health space, and the discussion has begun to focus on how to integrate the various elements and how to facilitate the development of mHealth platforms by coordinating the relationships between the players (10).

### 2.2. Health information quality

Regarding whether platforms will be able to attract and retain more users, the key question is whether they can provide high-quality health information. Quality health information needs to meet two criteria (24). First, it needs to be of high quality (25, 26). Second, the writing needs to be clear (27). Barry (28) was the first to explore a user-generated index system for evaluating the quality of web information. Eysenbach et al. (29) studied the literature related to the quality of health information on the Internet up to 2001 and derived the most commonly used criteria for evaluating the quality of health information on websites. These include accuracy, completeness, readability, site design, disclosure, and the reference(s) provided. However, the findings and conclusions concerning health-related websites vary widely due to differences in research methods, quality standards, study populations, and topic selection. Therefore, it is necessary to define a quality standard for health information. Zhang et al. (30) systematically analyzed 165 articles related to the quality of online health information from 2002 to 2013 and found that researchers typically evaluated quality in terms of content, format, and technical platform design. The quality of health information varies across medical fields and different websites, and overall information quality remains problematic. Future research needs to examine the quality of user-generated content and incorporate new media to facilitate consumer assessment of health information. Ewington et al. (31) systematically evaluated online health information regarding childbirth in terms of credibility, accuracy, readability, and content quality, and found that quality varied. Government websites have been found to have higher overall quality scores than non-government and commercially funded websites (32, 33), although there are significant differences between countries.

In terms of evaluating the quality of online health information, some scholars have used self-developed evaluation criteria, while others have directly used evaluation tools to rate the content attributes of health information. There are several popular tools for evaluating health websites, including the HON Code of Conduct for medical and health websites, issued by the Health on the Net Foundation (34); the DISCERN instrument, developed by the British Library at the University of Oxford and funded by the British National Health Service Executive Research and Development Program (35); the LIDA instrument, developed by Minervation in the UK healthcare field (36); and the JAMA instrument, created for the World Wide Web as a medical information delivery tool (37). In many cases, evaluation tools have been developed based on medical experts' input, which does not fully meet the needs of information users. Therefore, it is necessary to test the validity of the evaluation tools. It is generally agreed that online health information is poor in quality, so it is urgent that it should be improved. However, current research has mainly evaluated the quality of online health information, which is part of post-event supervision, lagging behind actual development, thereby posing a great danger in terms of faking health information. As a result, a monitoring mechanism for online health information is urgently needed, as well as a supervisory function before, during, and after the event.

### 2.3. Reputation mechanism

The reputation mechanism plays a central role in human societies and has aroused widespread interest in the realm of evolutionary game theory (38, 39). Reputation is seen as an important factor in building partnerships (40–42). Several behavioral experimental studies have demonstrated the important role of reputation in promoting cooperation (43). It is possible to use reputation as a signal to reveal the right direction for consumers, to reduce the degree of information asymmetry and the risk of adverse selection, to positively motivate the performance of physicians, and to improve the social influence and performance of platforms. Reputation enhancement can promote health knowledge sharing (44). The number of doctors' appointments is positively correlated with their reputation (45). Individuals with good reputations are able to earn more and they can influence the decisions of others, effectively promoting cooperation (46).

Providing quality health information on mHealth platforms is primarily the result of repeated games played by the providers and managers of health information content, aiming to maximize their own interests. This is a dynamic evolution relationship and matches the characteristics of the KMRW reputation model proposed by Kreps and Wilson (47). Thus, game theory is the most logical and explanatory analytical tool for analyzing reputation issues, and it also provides a quantitative measure of how reputation affects health information quality. Real-time evaluation of reputation levels of health information content providers and managers through a reputation mechanism is more effective than traditional post-event supervision in constraining their behavior and motivating them to provide high-quality services.

In conclusion, research on mHealth, network health information quality, reputation, and other aspects undertaken by domestic and foreign scholars has highlighted the significance of optimizing research on mHealth platforms' health information quality. At present, there are few studies on the quality of health information on mHealth platforms. Unlike previous studies, which have mainly focused on the content of the health information itself, the present study intends to introduce the reputation mechanism from the perspective of health information content providers and managers, construct an evolutionary game model, analyze the influencing factors of health information quality on mHealth platforms and of reputation on the optimization of health information quality, and investigate the sustainable development of mHealth platforms from the perspective of reputation.

## 3. Research methodology

### 3.1. Description of the research framework

According to evolutionary game theory, participants with limited rationality are unable to grasp fully their own powerful state due to information asymmetry and cognitive differences, etc., but can adjust their strategies by continuous learning, trial and error, imitation, and summarizing the experience of others. After repeated games, an equilibrium state is finally reached, which is the evolutionarily stable strategy (ESS). Neither providers nor managers of health information content on mHealth platforms are completely rational, and health information is essentially a trustworthy good that is difficult to judge accurately before use. It generally relies on the subjective reputation of information providers for judgment and decision-making. The quality of health information is actually a game between two groups of health information content providers and managers through random pairing and repeatedly adjusting their original strategies to maximize their own interests. This is consistent with the characteristics of evolutionary game theory, and the evolutionary game model can be constructed to analyze the health information quality of mHealth platforms. The framework model of this study was developed as follows:

1. According to the characteristics of health information content providers and managers on mHealth platforms, establish hypotheses and quantify the benefits of their strategies.
2. Construct a game pay-off matrix according to the hypotheses.
3. Analyze the stability of each strategy combination and its stability conditions.
4. Analyze the effect of each parameter change on the ESS.
5. Perform simulation analysis to verify the stability of the strategic combination.

In summary, due to information asymmetry, cognitive differences, and other factors, participants with bounded rationality may not fully understand their own powerful state. However, they may constantly learn, try again, copy, and sum up the experience of others to reach a stable equilibrium state, known as the ESS. The providers and managers of health information on mHealth platforms are not completely rational. Health information is essentially a trust item, which is difficult to evaluate accurately before using it. Usually, judgment and decision-making are based on a provider's subjective reputation (48). Essentially, health information quality is the result of providers and managers of health information content repeatedly playing through random pairings and continually tweaking their original strategies to maximize their own interests. The model developed in this study analyzes how health information quality can be optimized on mHealth platforms.

### 3.2. Model assumptions

Based on the characteristics of mHealth platform content providers and managers, the following assumptions are made:

#### 3.2.1. Assumption 1

Game subjects and their behavioral strategies. Physicians are the main providers of health information content on mHealth platforms. mHealth platforms, as the main providers and managers of health information content, play a crucial role in the quality of the health information provided. Physicians may spend a lot of time and energy editing their knowledge to publish and share high-quality health information on mHealth platforms, while others may publish and share low-quality health information due to a lack of professional knowledge, poor recognition skills, profit-driven, and limited time and energy. Thus, the physician's strategy choice space is (high quality, low quality). mHealth platforms, as the managers of health information quality, may actively fulfill their social responsibility by investing human, financial, and material resources to analyze, filter, encrypt, and desensitize sensitive information, thus actively controlling health information quality. As a result, the mHealth platform's strategy selection space is (control, not control).

#### 3.2.2. Assumption 2

Probability of adopting behavioral strategies for each subject. The game is asymmetric; in the initial state, the probability that the mHealth platform chooses the control strategy is *x* (0 ≤ *x* ≤ 1), and the probability that it chooses the no-control strategy is 1-*x*. The probability that physicians choose to provide high-quality health information is *y* (0 ≤ *y* ≤ 1), and the probability that they choose to provide low-quality health information is 1-*y*.

#### 3.2.3. Assumption 3

Benefits for health information providers. For physicians providing health information on the mHealth platform, the basic cost is *C*_*d*_ (such as editing their existing knowledge into health science articles, popular science videos, sharing classic cases, answering patients' questions, guiding medical treatment, etc.). The extra effort is *C*_*e*_ (for example, providing more accurate and credible health information). The basic gain obtained by the physician for providing health information is *E*_*d*_. When providing quality health information, the physician can obtain the reward *W* given by the patient and the reward *M* given by the mHealth platform, in addition to the reputation gain (*R*_1_). This gain (*R*_1_) is influenced by the reputation conversion coefficient α (α ≥ 0). The higher the reputation level of the physician, the greater the conversion factor α, and the greater the reputation benefits. The penalty suffered by the physician for providing low-quality health information when discovered by the health information manager is *P*. Further, the rent-seeking cost required to pay a gratuity to the manager in order to avoid the penalty may be *C*_*R*_, and the rent-seeking behavior is chosen with probability β (0 < β < 1).

#### 3.2.4. Assumption 4

Benefits for health information managers. The basic benefit of the mHealth platform to control the quality of health information is *E*_*p*_, and the cost to be paid is *C*_*p*_. The mHealth platform generates revenue *H* by providing high-quality health information. A physician's contribution rate *r* (0 ≤ *r* ≤ 1), i.e., the rate of income generated by increased investment in physician information quality, impacts this income. Furthermore, the control of the quality of health information by the mHealth platform can also bring about for itself a reputation benefit *R*_2_, which is affected by its reputation conversion coefficient η (η ≥ 0). If doctors provide low-quality health information and the mHealth platform does not control it, the platform is exposed with a probability of *b* (0 ≤ *b* ≤ 1) and suffers a loss *L*.

### 3.3. Model building

Based on the above assumptions, the behavioral choice and game payment matrix for the “provider–manager” of health information on mHealth platforms is constructed (see Table 1).

**Table 1:** Behavioral choices and game payment matrix for health information providers and managers of mHealth platforms.

**Physician (provider)**	**mHealth platform (administrator)**	
	**Control**	**No control**
High quality	*E*_*d*_+*W*+*M*+α*R*_1_−*C*_*d*_−*C*_*e*_	*E*_*d*_+*W*+α*R*_1_−*C*_*d*_−*C*_*e*_
	*E*_*p*_+*H*+η*R*_2_−*C*_*p*_	*rH*+(1−*b*)η*R*_2_
Low quality	*E*_*d*_−*C*_*d*_−(1−β)*P*−β(*C*_*R*_−*P*)	*E*_*d*_−*C*_*d*_−*bP*+(1−*b*)α*R*_1_
	*E*_*p*_+η*R*_2_+β*C*_*R*_−*C*_*p*_	−*bL*

Let the expected benefit of mHealth platforms choosing to control the quality of health information be *E*_*x*_:


$$E_{x}=y(E_{p}+H+\eta R_{2}-C_{p})+(1-y)(E_{p}+\eta R_{2}\text{+}\beta C_{R}-C_{p})$$

Let the expected benefit of mHealth platforms choosing not to control the quality of health information be *E*_1-*x*_:


$$E_{1\text{-}x}\text{=}y(rH+(1-b)\eta R_{2})+(1-y)(-bL)$$

Then, the average expected return of mHealth platforms is $\bar{E_{1}}$:


$$\begin{array}{l} \bar{E_{1}}=xE_{x}+(1-x)E_{1-x}\\ \;=x[y(E_{p}+Hn+\eta R_{2}-C_{p})+(1-y)\\ \;(E_{p}+\eta R_{2}+\beta C_{R}-C_{p})]\\ \;+(1-x)[y(rH+(1-b)\eta R_{2})+(1-y)(-bL)] \end{array}$$

Similarly, the expected benefits of physicians choosing to provide high-quality health information is *E*_*y*_:


$$\begin{array}{l} E_{y}=x(E_{d}+W+M+\alpha R_{1}-C_{d}-C_{e})\\ \;+(1-x)(E_{d}+W+\alpha R_{1}-C_{d}-C_{e}) \end{array}$$

The expected benefits of physicians choosing to provide low-quality health information is *E*_1-*y*_:


$$\begin{array}{l} E_{1\text{-}y}=x(E_{d}-C_{d}-(1-\beta )P-\beta (C_{R}-P))\\ \;+(1-x)(E_{d}-C_{d}-bP+(1-b)\alpha R_{1}) \end{array}$$

The average expected return for physicians is $\bar{E_{2}}$:


$$\begin{array}{l} \bar{E_{2}}=yE_{y}+(1-y)E_{1-y}\\ \;=y[x(E_{d}+W+M+\alpha R_{1}-C_{d}-C_{e})+(1-x)\\ \;(E_{d}+W+\alpha R_{1}-C_{d}-C_{e})]\\ \;+(1-y)[x(E_{d}-C_{d}-(1-\beta )P-\beta (C_{R}-P))\\ \;+(1-x)(E_{d}-C_{d}-bP+(1-b)\alpha R_{1})] \end{array}$$

Thus, the replication dynamic equations for the mHealth platform and physician strategy are then obtained:


$$\begin{array}{l} F(x)=dx/dt=x(E_{x}-\bar{E_{1}})\\ \;=x(1-x)[y[(1-r)H-\beta C_{R}-(1-b)\eta R_{2}-bL]\\ \;+E_{p}+\eta R_{2}+\beta C_{R}+bL-C_{P}] \end{array}$$

$$\begin{array}{l} F(y)=dy/dt=y(E_{y}-\bar{E_{2}})\\ \;=y(1-y)[x[\beta (C_{R}-P)-(\beta +b)P+(1-b)\\ \;\alpha R_{1}+M+P]+W-C_{e}+bP+b\alpha R_{1}] \end{array}$$

### 3.4. Stability analysis of evolutionary strategies

The health information manager–provider game system is obtained from the replicated dynamic equations *F*(*x*) and*F*(*y*). There are five partial equilibrium points in the plane *M* = {(*x, y*)|0 ≤ *x* ≤ 1, 0 ≤ *y* ≤ 1}. They are *E*_1_(0, 0), *E*_2_(0, 1), *E*_3_(1, 0), *E*_4_(1, 1), and $E_{5}\left(x^{*},y^{*}\right)$, where:


$$\begin{array}{c} x^{*}=\frac{C_{e}-W-bP-b\alpha R_{1}}{\beta \left(C_{R}-P\right)-\left(\beta +b\right)P+\left(1-b\right)\alpha R_{1}+M+P} \end{array}$$

$$\begin{array}{c} y^{*}=\frac{C_{p}-E_{p}-\eta R_{2}-\beta C_{R}-bL}{\left(1-r\right)H-\beta C_{R}-\left(1-b\right)\eta R_{2}-bL} \end{array}$$

These equalization points are not necessarily ESSs for the system. ESSs have the ability to prevent invasion and disturbance, as they are subject to bounded rationality that continuously adjusts the strategy according to the vested interests in terms of pursuing their own interests, eventually achieving a dynamic balance. According to the method proposed by Friedman, the stability of the equilibrium point of the evolutionary system of health information managers and providers can be verified by the local stability analysis of the Jacobi matrix of the system based on the replicated dynamic equations *F*(*x*) and *F*(*y*), and the Jacobi matrix of the system *J*:


$$\begin{array}{c} J=\left[\begin{array}{c} J_{1},J_{2}\\ J_{3},J_{4} \end{array}\right] \end{array}$$

$$\begin{array}{l} J_{1}=(1-2x)[y\left[(1-r)H-\beta C_{R}-(1-b)\eta R_{2}-bL\right]\\ \;+E_{p}+\eta R_{2}+\beta C_{R}+bL-C_{p}] \end{array}$$

$$\begin{array}{c} J_{2}=x\left(1-x\right)\left[\left(1-r\right)H-\beta C_{R}-\left(1-b\right)\eta R_{2}-bL\right] \end{array}$$

$$\begin{array}{c} J_{3}=y\left(1-y\right)\left[\beta \left(C_{R}-P\right)-\left(\beta +b\right)P+\left(1-b\right)\alpha R_{1}+M+P\right] \end{array}$$

$$\begin{array}{l} J_{4}=(1-2y)[x\left[\beta (C_{R}-P)-(\beta +b)P+(1-b)\alpha R_{1}+M+P\right]\\ \;+W-C_{e}+bP+b\alpha R_{1}] \end{array}$$

When the equilibrium point satisfies the Jacobi matrix determinants *detJ* > 0 and *trJ* < 0, the equilibrium point is replicating the ESS of the dynamical system.

Since the parameter values cannot be determined, the stability of the equilibrium point needs to be further explored in a categorical manner. When conditions satisfy:


$$\begin{array}{c} 0\leq \frac{C_{e}-W-bP-b\alpha R_{1}}{\beta \left(C_{R}-P\right)-\left(\beta +b\right)P+\left(1-b\right)\alpha R_{1}+M+P}\leq 1 \end{array}$$

and


$$\begin{array}{c} 0\leq \frac{C_{p}-E_{p}-\eta R_{2}-\beta C_{R}-bL}{\left(1-r\right)H-\beta C_{R}-\left(1-b\right)\eta R_{2}-bL}\leq 1 \end{array}$$

the following four cases can be classified according to the comparison of the gain conditions at the time of strategy selection between health information providers and managers.

#### 3.4.1. Case 1

When *E*_*p*_ + η*R*_2_ + β*C*_*R*_ − *C*_*p*_ > −*bL*, *W* − *C*_*e*_ + *bαR*_1_ > − *bP*, and when α*R*_1_ + *M* + *W* − *C*_*e*_ + β(*C*_*R*_−*P*) + (1 −β)*P*>0, *E*_*p*_−*C*_*p*_ + (1 − *r*)*H* + *bηR*_2_ > 0; after the dynamic evolutionary game, the net benefits both of the health information providers and the managers of the mHealth platform are positive, and their evolutionary stability points are *E*_4_(1, 1). A physician will provide high-quality health information when the benefits of doing so outweigh the benefits of providing low-quality health information, regardless of whether the mHealth platform controls the quality of health information. In the same way, when controlling the quality of health information is more beneficial than not controlling it, regardless of whether doctors provide high-quality health information, the mHealth platform will decide to control it.

#### 3.4.2. Case 2

When *E*_*p*_ + η*R*_2_ + β*C*_*R*_ − *C*_*p*_ > −*bL*, *W* − *C*_*e*_ + *bαR*_1_ < −*bP*, and when α*R*_1_ + *M* + *W* − *C*_*e*_ + β(*C*_*R*_ − *P*) + (1 − β)*P* > 0, *E*_*p*_ − *C*_*p*_ + (1 − *r*)*H* + *bηR*_2_> 0; after the dynamic evolutionary game, the mHealth platform has a positive net benefit and the physician has a negative net benefit. Its evolutionary stability point is *E*_4_(1, 1). Physicians commit a lot of time and effort to publishing and sharing high-quality health information when the mHealth platform does not control the quality. If physicians have a good reputation, the system will eventually form a positive relationship. mHealth platforms will eventually reach equilibrium when they control the quality of health information, regardless of whether the physician group provides high-quality information. If doctors initially provide low-quality health information, they will be punished under the condition that the mHealth platform actively monitors the quality of health information, and the punishment is greater than the rent-seeking cost paid by physicians to avoid punishment. Therefore, the system will eventually form a positive relationship by providing high-quality health information.

#### 3.4.3. Case 3

When *E*_*p*_ + η*R*_2_ + β*C*_*R*_ − *C*_*p*_ < − *bL*, *W* − *C*_*e*_ + *bαR*_1_ < − *bP*, and when α*R*_1_ + *M* + *W* − *C*_*e*_ + β(*C*_*R*_ − *P*) + (1 − β)*P*> 0, *E*_*p*_ − *C*_*p*_ + (1 − *r*)*H* + *bηR*_2_ > 0; after the dynamic evolutionary game, the mHealth platform and the physician both have a negative net benefit. Its evolutionary stability point is *E*_1_(0, 0) or *E*_4_(1, 1). When physician groups provide low-quality health information, mHealth platforms gain less by controlling the quality of health information than they lose by being exposed when they do not. When mHealth platforms do not control the quality of health information, the benefits of providing high-quality health information from physicians are less than the benefits of being exposed and penalized for providing low-quality health information. In such a situation, the system will eventually evolve into and end up in a prisoner's dilemma. Nevertheless, the interaction between the mHealth platform and doctors will eventually become positive as the platform controls the quality of health information and doctors provide high-quality health information.

#### 3.4.4. Case 4

When *E*_*p*_+η*R*_2_+β*C*_*R*_−*C*_*p*_ < −*bL*, *W*−*C*_*e*_+*bαR*_1_>−*bP*, and when α*R*_1_+*M*+*W*−*C*_*e*_+β(*C*_*R*_−*P*) + (1−β)*P*>0, *E*_*p*_−*C*_*p*_+(1−*r*)*H*+*bηR*_2_>0; after the dynamic evolutionary game, the mHealth platform has a negative net benefit and the physician has a positive net benefit. Its evolutionary stability point is *E*_4_(1, 1). In the case of physician groups providing low-quality health information, the mHealth platform must invest in costs in order to control the quality of that information, resulting in a negative net income for the platform. The system will eventually evolve to a benign state due to the good reputation of the platform. Ultimately, the system will evolve to an equilibrium state if physician groups provide high-quality health information, regardless of whether the mHealth platform controls the quality of health information. This is due to the fact that if the mHealth platform does not control the quality of health information when low-quality health information provided by doctors is exposed, the loss is much greater than the short-term benefits for the platform. In the long run, mHealth platforms will choose to control the quality of health information so that the system will eventually interact in a positive way.

According to the local stability analysis method of the Jacobi matrix, the system equilibrium point (evolutionary stability) results were obtained (see Table 2).

**Table 2:** Results of local stability analysis.

**Equilibrium points**	**Case 1**			**Case 2**			**Case 3**			**Case 4**		
	***detJ***	***trJ***	**Stable**	***detJ***	***trJ***	**Stable**	***detJ***	***trJ***	**Stable**	***detJ***	***trJ***	**Stable**
*E*_1_(0, 0)	+	+	Unstable	–	Uncertain	Saddle point	+	–	ESS	–	Uncertain	Saddle point
*E*_2_(0, 1)	–	Uncertain	Saddle point	+	+	Unstable	+	+	Unstable	–	Uncertain	Saddle point
*E*_3_(1, 0)	–	Uncertain	Saddle point	–	Uncertain	Saddle point	+	+	Unstable	+	+	Unstable
*E*_4_(1, 1)	+	–	ESS	+	–	ESS	+	–	ESS	+	–	ESS
$E_{5}\left(x^{*},y^{*}\right)$	Uncertain	0	Center or saddle point	Uncertain	0	Center or saddle point	Uncertain	0	Center or saddle point	Uncertain	0	Center or saddle point

From Table 2, it can be seen that when the reputation mechanism and punishment mechanism are considered, providing high-quality health information from physicians is more beneficial than providing low-quality health information, and controlling the quality of health information is more beneficial than having no control when the health information quality improves. The equilibrium points of system evolution are all in Cases 1–4. In other words, excluding the extra cost of providing high-quality health information, the benefits for physicians (including reputation gains, incentives given to physicians by the platform, and incentives given to physicians by patients) are greater than the penalties suffered by physicians for providing low-quality health information (the rent-seeking costs incurred to avoid the penalties). Further, the benefits of actively controlling the quality of health information by the mHealth platform (including reputation gain) are greater than the opportunity benefits gained from not controlling the quality of health information without exposure (including revenue and reputation benefits to the platform from quality information provided by physicians). In this case, both parties will adopt the strategy of providing high-quality health information and the manager will adopt the strategy of controlling the quality of health information after a period of game play as long as the benefits of one party not controlling the quality of health information are less than the benefits when controlling. The Jacobi matrix determinant columns *detJ* > 0 and *trJ* < 0 of the equilibrium points *E*_2_(0, 1) and *E*_3_(1, 0) are not constant and therefore cannot be an ESS for the system. As a result, health information managers and providers on mHealth platforms unilaterally optimizing the quality of health information is unreliable.

**Figure 1:**
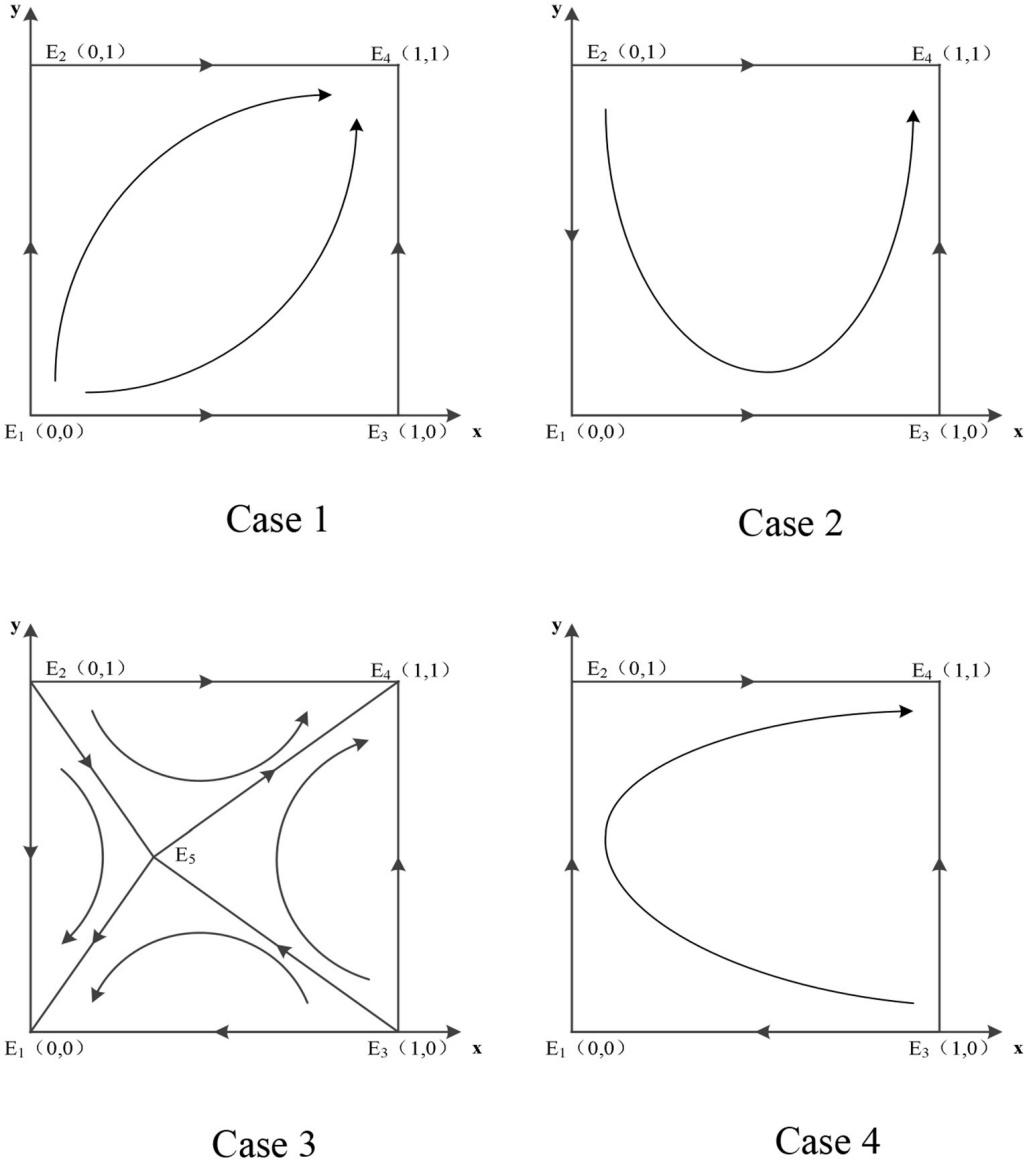
Evolutionary phase diagram of the system under different cases.

By varying each parameter's size, the evolution phase diagrams of the system are obtained (see Figure 1), allowing a more intuitive understanding of the evolution path.

### 3.5. Effect of parameter changes on evolutionarily stable strategies

When there are two possible equilibria of the system, changes in the magnitude of different parameter values cause the system to converge to different equilibria, as in Case 3. Both point *E*_1_(0, 0) and point *E*_4_(1, 1) are ESSs of the system, and whether the system converges to *E*_1_(0, 0) or *E*_4_(1, 1) requires further analysis depends on the variation of the parameters. From the phase diagram of the system evolution in Case 3 in Figure 1, it is clear that the key to the system evolution results lies in the position where the central point or saddle point *E*_5_ is located. The changes of the relevant parameters will lead to the movement of *E*_5_, thus playing the role of regulating the evolution direction. The effects of the changes of each parameter on the system evolution results are shown as follows:

(1) Parameter *C*_*e*_. *C*_*e*_ is the additional cost for physicians to provide quality health information.. As *C*_*e*_ increases, *E*_5_ moves horizontally to the right, and the area of region *E*_1_*E*_3_*E*_5_*E*_2_ expands. Eventually, the system becomes more likely to converge to *E*_1_(0, 0). To put it another way, too much energy spent on providing high-quality health information will negatively affect physicians' enthusiasm, which will harm the system's benign evolution.

(2) Parameter *W*, *M*. *W* is the reward given by the patient when the physician provides quality health information. As *W* increases, *E*_5_ moves horizontally to the left and the area of region *E*_2_*E*_5_*E*_3_*E*_4_ expands. Eventually, the system becomes more likely to converge to *E*_4_(1, 1), indicating that patient affirmation facilitates physicians to provide quality health information. *M* is the reward given by the mHealth platform when physicians provide quality health information. As *M* increases, *E*_5_ moves horizontally to the left and the system becomes more likely to converge to *E*_4_(1, 1), indicating that incentives on mHealth platforms facilitate physicians' delivery of quality health information. The more rewards health information providers receive, the more conducive they are to the benign evolution of the system.

(3) Parameter *R*_1_, α, *R*_2_, η. *R*_1_ is the reputation gain that physicians receive when they provide quality information. As *R*_1_ increases, *E*_5_ moves horizontally to the left and the area of region *E*_2_*E*_5_*E*_3_*E*_4_ expands. Eventually, the system becomes more likely to converge to *E*_4_(1, 1). α is the conversion factor for physicians to provide quality information for reputation gain. As α increases, *E*_5_ moves horizontally to the left and the area of region *E*_2_*E*_5_*E*_3_*E*_4_ expands. Eventually, the system becomes more likely to converge to *E*_4_(1, 1). It can be seen that the parameters *R*_1_ and α are changing in the same direction in the system. *R*_2_ is the reputation benefit gained when the mHealth platform controls the quality of health information. As *R*_2_ increases, *E*_5_ moves vertically downward and the area of region *E*_2_*E*_5_*E*_3_*E*_4_ expands. Eventually, the system becomes more likely to converge to *E*_4_(1, 1).

η is the conversion coefficient of the mHealth platform to control the quality of health information for reputation gain. As η increases, *E*_5_ moves vertically downward and the area of region *E*_2_*E*_5_*E*_3_*E*_4_ expands, contributing to the benign evolution of the system. It can be seen that the parameters *R*_2_ and η are changing in the same direction in the system.

This shows that reputation mechanisms can motivate physicians to provide quality health information and monitor the quality of health information managed by mHealth platforms.

(4) Parameter *P*, β, *C*_*R*_. *P* is the penalty that physicians suffer when they provide low-quality health information that is detected by health information managers. When *P* increases, the value of y^*^ is constant and the value of x^*^ is influenced by the probability β of physicians choosing rent-seeking behavior. When $\text{0}<\beta {<}^{1}{/}_{2}$, the value of x^*^ becomes smaller, and *E*_5_ shifts horizontally to the left, making the area of the region *E*_2_*E*_5_*E*_3_*E*_4_ larger. Thus, the possibility of the system evolving to a stable point becomes greater. When 12<β<1, the change in the value of x^*^ cannot be determined, the impact on the quality of health information on the mobile over medical platform cannot be judged at this time. As β grows, y^*^ becomes smaller, and x^*^ is affected by *P* and the rent-seeking cost *C*_*R*_.

When $P{<}^{C_{R}}{/}_{2}$, x^*^ becomes smaller, and *E*_5_ moves to the lower left corner, thereby expanding the region *E*_2_*E*_5_*E*_3_*E*_4_, which is conducive to the benign evolution of the system. When $P{>}^{C_{R}}{/}_{2}$, x^*^ becomes bigger; it is impossible to determine how the area of *E*_2_*E*_5_*E*_3_*E*_4_ changes, so the influence of β on the quality of the mHealth platform's health information cannot be judged. As the rent-seeking cost *C*_*R*_ increases, the saddle point *E*_5_ moves horizontally to the left and vertically downward, and the area *E*_1_*E*_3_*E*_5_*E*_2_ where the system converges to the bad mode becomes smaller, which is beneficial to its benign evolution. The greater the rent-seeking cost, the more willing physicians are to provide quality health information.

(5) Parameter *E*_*p*_, *C*_*p*_. *E*_*p*_ is the revenue generated by physicians providing high-quality health information to mHealth platforms. As *H* increases, *E*_5_ moves vertically downward and the area of region *E*_2_*E*_5_*E*_3_*E*_4_ expands, contributing to the benign evolution of the system.

*E*_*p*_ is the basic benefit of mHealth platforms for controlling the quality of health information. As *E*_*p*_ increases, *E*_5_ moves vertically downward and the area of region *E*_2_*E*_5_*E*_3_*E*_4_ expands, contributing to the benign evolution of the system. *C*_*p*_ is the cost that mHealth platforms need to pay to control the quality of health information. As *C*_*p*_ increases, *E*_5_ moves vertically upward and the area of region *E*_1_*E*_3_*E*_5_*E*_2_ expands, thus not contributing to the benign evolution of the system.

(6) Parameter *H*,*r*. *H* is the revenue generated by physicians providing high-quality health information to mHealth platforms. As *H* increases, *E*_5_ moves vertically downward and the area of region *E*_2_*E*_5_*E*_3_*E*_4_ expands, contributing to the benign evolution of the system. *r* is the contribution of physician input. As *r* increases, *E*_5_ moves vertically upward and the area of region *E*_2_*E*_5_*E*_3_*E*_4_ expands, thus not being conducive to the benign evolution of the system. This shows that if mHealth platforms are not under control, no matter how much physicians invest, health information cannot be guaranteed to be of high quality.

(7) Parameter *b*, *L*. *b* reflects mHealth platforms choosing not to control the probability of being exposed when physicians provide low-quality health information. As *b* increases, the saddle point *E*_5_ moves horizontally to the left and vertically downward at the same time. The area of region *E*_2_*E*_5_*E*_3_*E*_4_ expands, contributing to the benign evolution of the system. *L* is the loss suffered by mHealth platforms due to the exposure of low-quality health information. As *L* increases, *E*_5_ moves vertically downward and the area of region *E*_2_*E*_5_*E*_3_*E*_4_ expands, contributing to the benign evolution of the system.

## 4. Simulation analysis

For a more intuitive analysis of the dynamic evolution process of the health information providers and managers of mHealth platforms, and to verify that the evolutionary game model above is valid, we use the MATLAB simulation tool to run numerical simulations for different situations and analyze their effects according to the constraints and copy the dynamic equations.

### 4.1. Case 1

**Figure 2:**
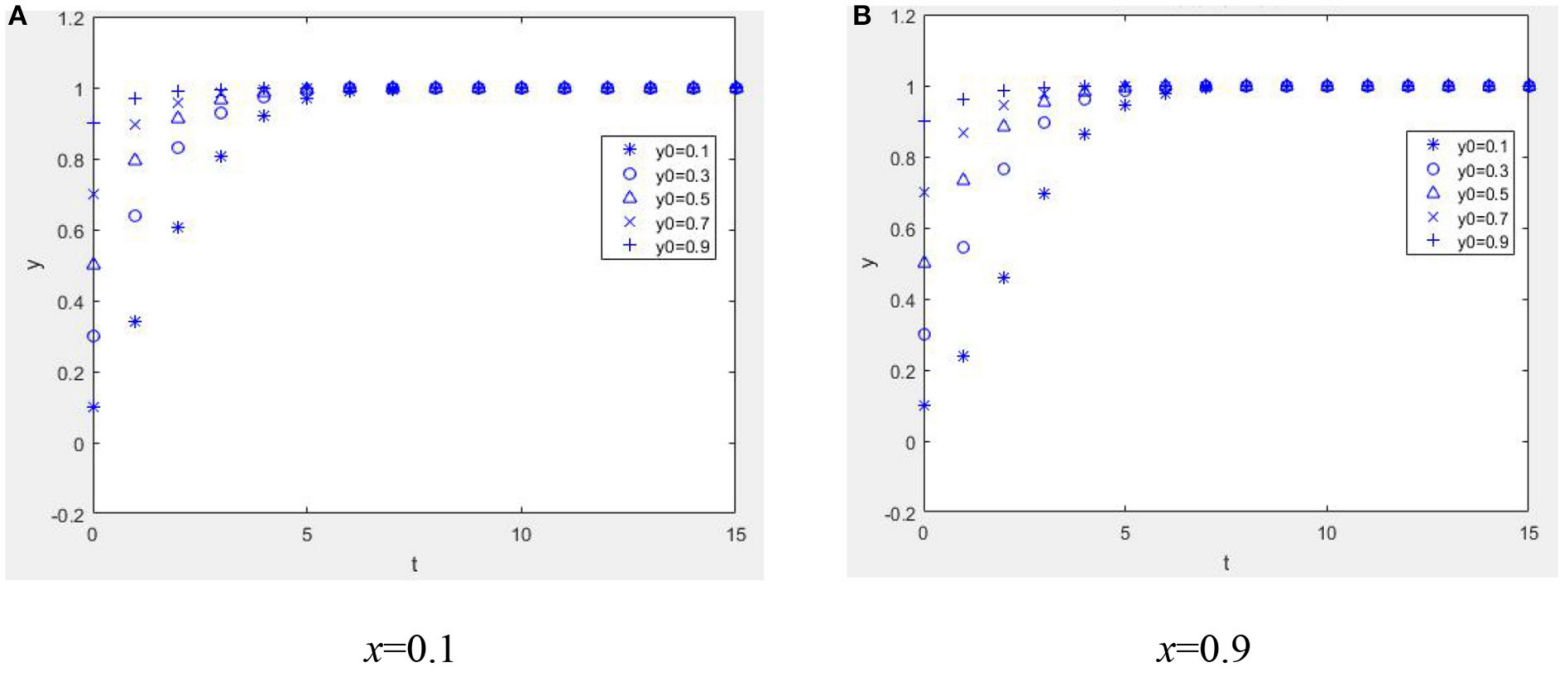
Dynamc evolution diagram under Case 1. **(A)** *x* = 0.1 and **(B)** *x* = 0.9.

Suppose *W* = 1, *M* = 1, α = 2, *R*_1_ = 2, *C*_*e*_ = 1, *E*_*p*_ = 1, *C*_*p*_ = 1, *H* = 1, *r* = 0.2, *R*_2_ = 1, η = 1, *b* = 0.2, *L* = 2, β = 0.2, *C*_*R*_ = 2, and *P* = 4; when *E*_*p*_+η*R*_2_+β*C*_*R*_−*C*_*p*_>−*bL*, *W*−*C*_*e*_+*bαR*_1_>−*bP* and when α*R*_1_ + *M* + *W* − *C*_*e*_ + β(*C*_*R*_ − *P*) + (1 − β)*P* > 0, *E*_*p*_ − *C*_*p*_ + (1 − *r*)*H* + *bηR*_2_ > 0. From Figure 2, it can be seen that the equilibrium point of the game between the health information providers and managers of the mHealth platform is (1, 1). Figure 2A illustrates that as long as the benefit of the mHealth platform controlling the quality of health information is greater than the benefit of not controlling the quality of health information, no matter what percentage of physicians initially choose to provide high-quality health information, health information managers actively monitor the quality of health information. As seen in Figures 2A, B, if the benefits of providing high-quality health information outweigh the benefits of providing low-quality health information, regardless of the proportion of the strategy initially chosen by the mHealth platform to control the quality of health information, physician groups who are rational economic people will actively provide high-quality health information.

### 4.2. Case 2

**Figure 3:**
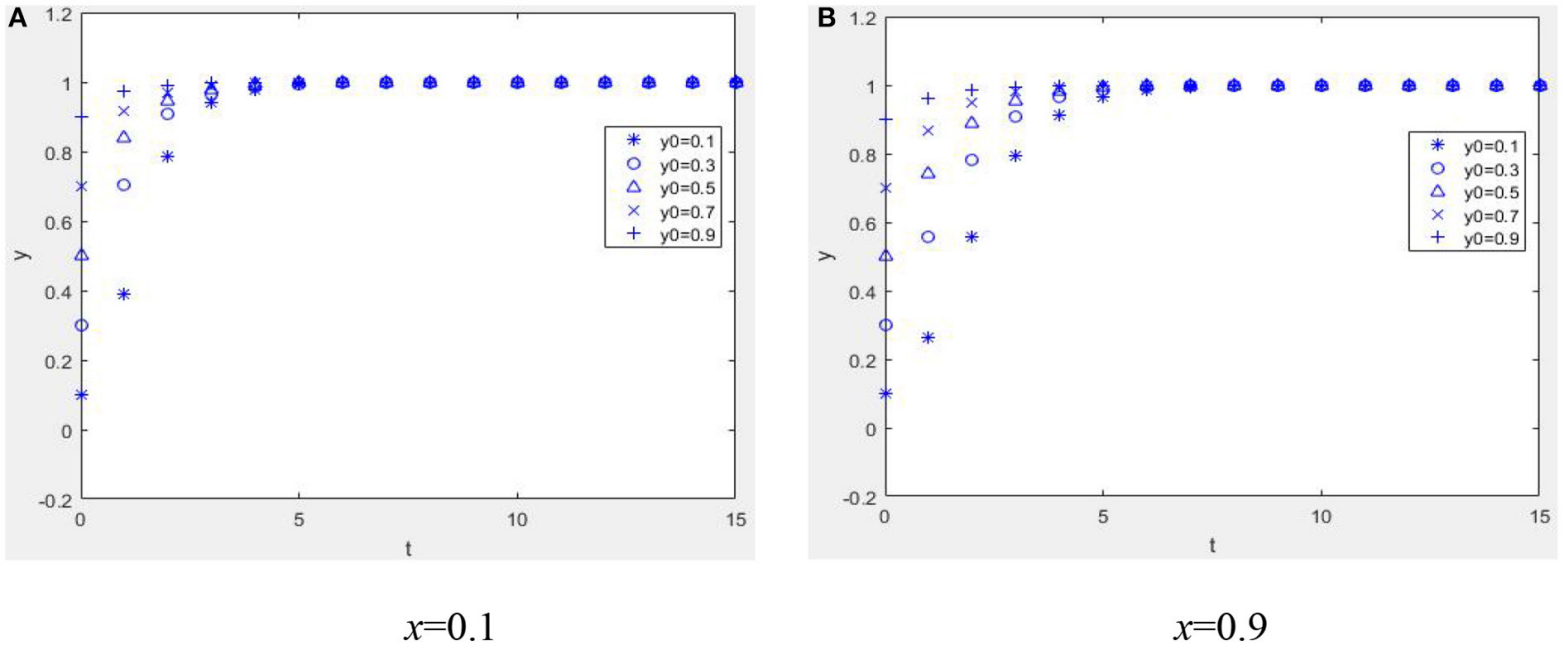
Dynamic evolution diagram under Case 2. **(A)** *x* = 0.1 and **(B)** *x* = 0.9.

Suppose *W* = 1, *M* = 1, α = 2, *R*_1_ = 2, *C*_*e*_ = 5, *E*_*p*_ = 1, *C*_*p*_ = 1, *H* = 1, *r* = 0.2, *R*_2_ = 1, η = 1, *b* = 0.2, *L* = 2, β = 0.2, *C*_*R*_ = 2, and *P* = 4; when *E*_*p*_ + η*R*_2_ + β*C*_*R*_ − *C*_*p*_ > −*bL*, *W* − *C*_*e*_ + *bαR*_1_ < −*bP* and when α*R*_1_ + *M* + *W* − *C*_*e*_ + β(*C*_*R*_ − *P*) + (1 − β)*P* > 0, *E*_*p*_ − *C*_*p*_ + (1 − *r*)*H* + *bηR*_2_ > 0. From Figure 3, it can be seen that the equilibrium point of the game between health information providers and managers of the mHealth platform is (1, 1). Figure 3A shows that when the mHealth platform's proportion of strategies to control quality is low, the more physicians choose strategies to provide high-quality health information, and the faster the time spent reaching equilibrium, i.e., the more physicians invest in the system's benign evolution, the more favorable it is for the reputation mechanism of physicians to play a positive role. Figure 3B shows that if the mHealth platform chooses a high proportion of strategies to manage and control the quality of health information, no matter whether the physician group chooses a high or low proportion of strategies, the system will evolve to an equilibrium point. The time to reach the equilibrium point is different, and the punishment mechanism of the mHealth platform is beneficial.

### 4.3. Case 3

**Figure 4:**
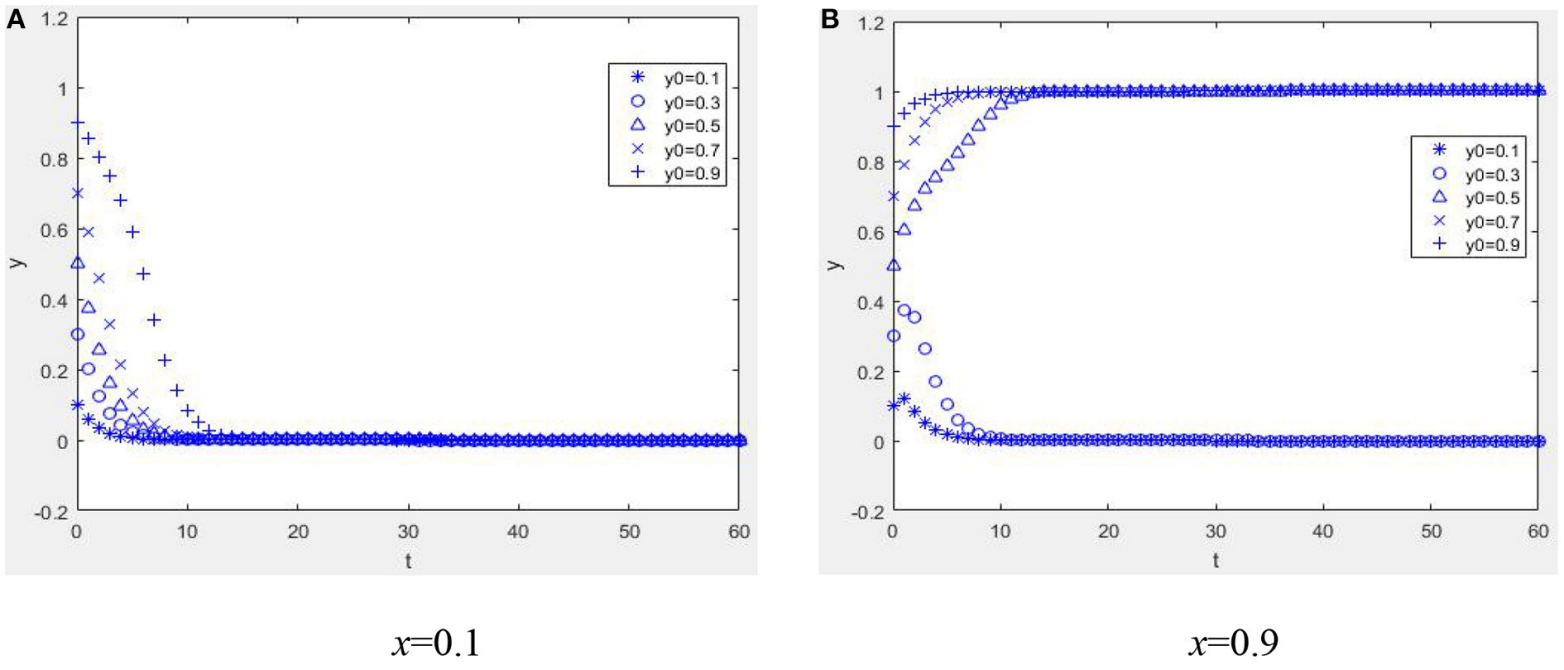
Dynamic evolution diagram under Case 3. **(A)** *x* = 0.1 and **(B)** *x* = 0.9.

Suppose *W* = 1, *M* = 1, α = 2, *R*_1_ = 2, *C*_*e*_ = 5, *E*_*p*_ = 1, *C*_*p*_ = 3, *H* = 3, *r* = 0.2, *R*_2_ = 1, η = 1, *b* = 0.2, *L* = 2, β = 0.2, *C*_*R*_ = 0.1, and *P* = 0.04; when *E*_*p*_ + η*R*_2_ + β*C*_*R*_ − *C*_*p*_ < −*bL*, *W* − *C*_*e*_ + *bαR*_1_ < −*bP* and when α*R*_1_ + *M* + *W* − *C*_*e*_ + β(*C*_*R*_ − *P*) + (1 − β)*P* > 0, *E*_*p*_ − *C*_*p*_ + (1 − *r*)*H* + *bηR*_2_ > 0. As shown in Figure 4A, when the mHealth platform and physician group both have negative net incomes, the equilibrium point between the mHealth platform and manager is (1, 1). When the mHealth platform has a high proportion of strategies to control the quality of health information (*x* = 0.9, other parameters taken as in Figure 4A, compare Figures 4A,B), it is possible to evolve the system to the equilibrium point (1, 1), which validates the previous theoretical analysis.

**Figure 5:**
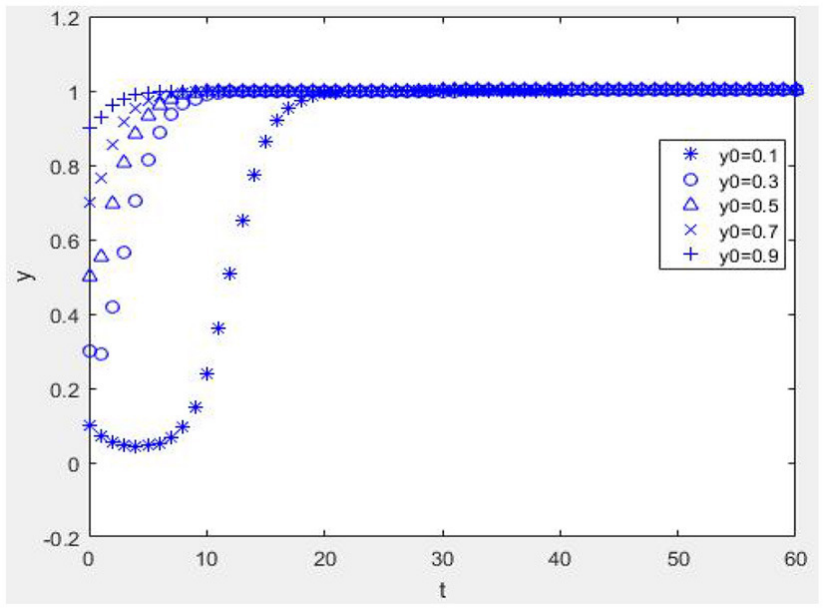
Dynamic evolution diagram under Case 3(*R*_1_ = 9 or α = 9).

Except for these parameters, other parameters take the same values as Figure 4A, and by comparing Figures 4A, 5 it can be seen that when a reputation gain is obtained (*R*_1_ = 9), the system can achieve benign evolution even if the mHealth platform does not have a high proportion of strategies to control the quality of health information. Also, the higher the proportion of physicians providing quality information, the shorter the time to converge to the equilibrium state. According to this study, when the control ratio of the mHealth platform is low, the reputation mechanism can positively guide the behavior of health information providers, prompting them to provide high-quality health information, which is conducive to the system's benign evolution. In the system, the parameters *R*_1_ and α change in the same direction. When the other parameters are the same as in Figure 4A and α = 9, the dynamic evolution diagram is the same in Figure 5. When the conversion coefficient of high-quality information to obtain reputation benefits increases, it is conducive to the benign evolution of the system. When the conversion coefficient of reputation gain from physicians providing quality information becomes low [e.g., Figure 4A, α = 2], it is not conducive to the benign evolution of the system. However, the quality of health information can still be guaranteed when the control ratio of mHealth platforms is high [e.g., Figure 4B, *x* = 0.9] and the proportion of physician groups providing quality health information is also high. This shows that when reputation mechanisms play a minor role, it takes the combined efforts of mHealth platforms and physician groups to evolve the system to a benign state.

**Figure 6:**
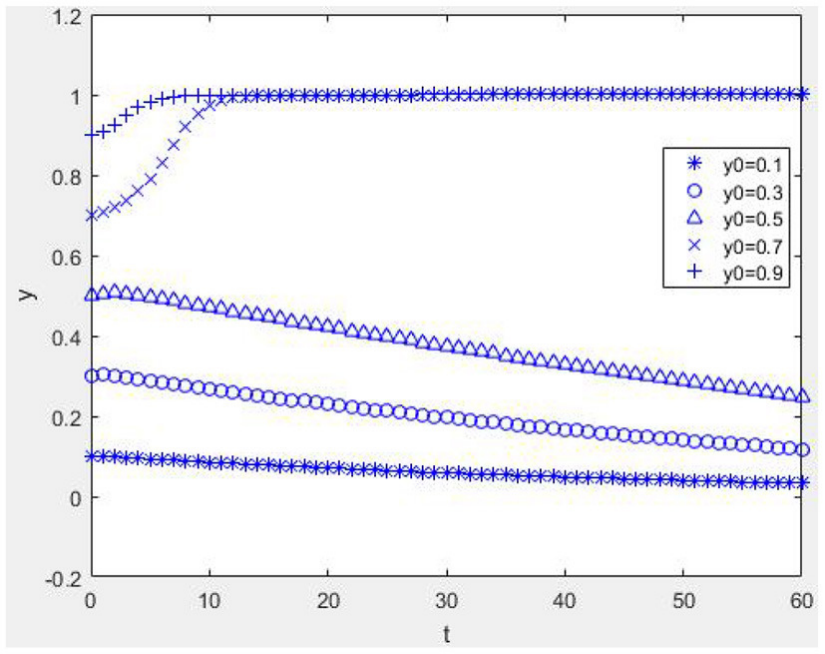
Dynamic evolution diagram under Case 3(*C*_*R*_ = 2.9).

From Figure 4A, we can see that the role of the reputation mechanism is not significant due to the small reputation gain and reputation conversion coefficient. When the rent-seeking cost of the physician population increases [except for the parameter *C*_*R*_ = 2.9, other parameters take the same values as Figure 4A, compare Figures 4A, 6], it is conducive to the benign evolution of the system, indicating that the increase in the rent-seeking cost of physicians is conducive to reducing the rent-seeking behavior of physicians and motivating them to provide quality health information.

**Figure 7:**
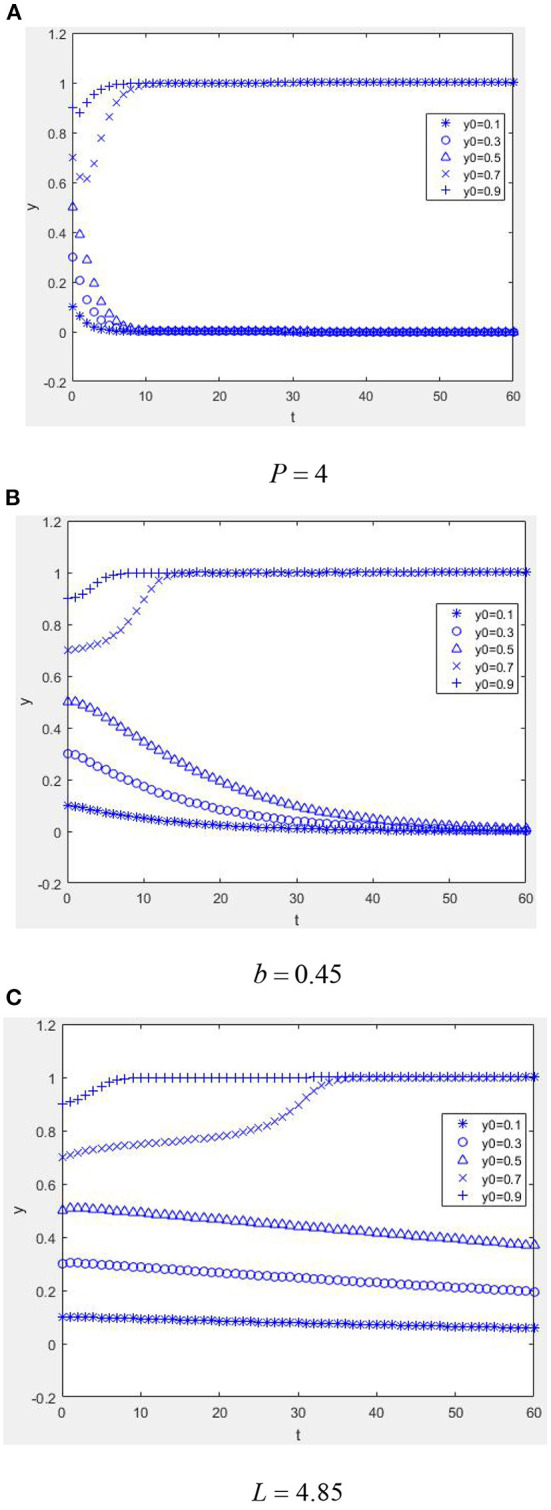
Dynamic evolution diagram under Case 3 (the role of punishment mechanism). **(A)** *P* = 4, **(B)** *b* = 0.45, and **(C)** *L* = 4.85.

It is conducive to the benign evolution of the health information system when physicians provide low-quality health information and are punished for it (except for parameter *P* = 4, other parameters take the same values as in Figure 4A, compare Figures 4A, 7A). In situations where physicians provide low-quality health information (except for parameter *b* = 0.45, other parameters take the same values as in Figure 4A, compare Figures 4A, 7B), it is conducive to the system's benign evolution. An increase in the loss suffered by the mHealth platform due to low-quality health information is conducive to a benign evolution of the system (except for the parameter *L* = 4.85, other parameters take the same values as Figure 4A, compare Figures 4A, 7C), which indicates that a reasonable punishment mechanism is necessary for the optimization of the platform's health information quality.

### 4.4. Case 4

**Figure 8:**
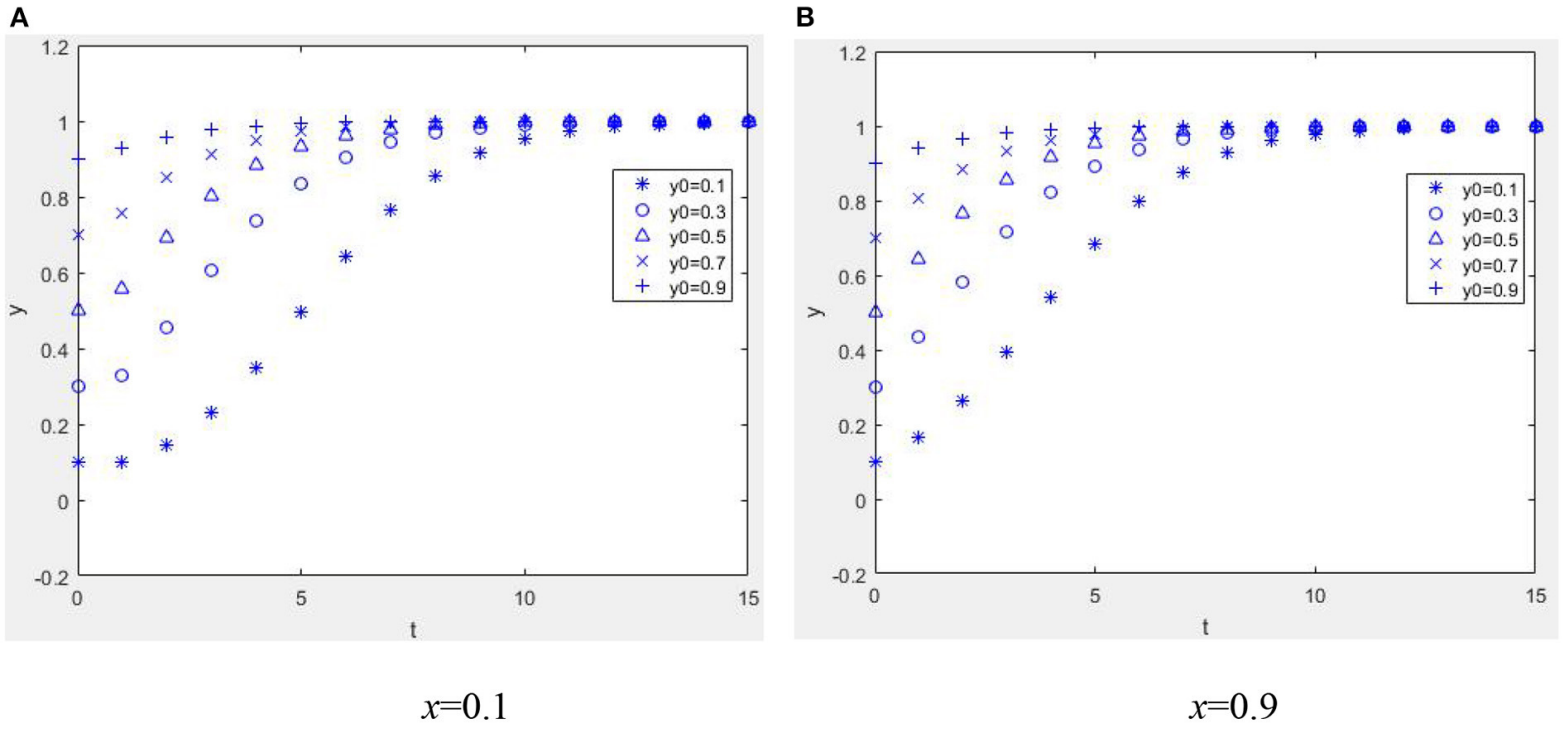
Dynamic evolution diagram under Case 4. **(A)** *x* = 0.1 and **(B)** *x* = 0.9.

Suppose *W* = 1, *M* = 1, α = 2, *R*_1_ = 2, *C*_*e*_ = 1, *E*_*p*_ = 1, *C*_*p*_ = 3, *H* = 3, *r* = 0.2, *R*_2_ = 1, η = 1, *b* = 0.2, *L* = 2, β = 0.2, *C*_*R*_ = 2, and *P* = 4; when *E*_*p*_ + η*R*_2_ + β*C*_*R*_ − *C*_*p*_ < −*bL*, *W* − *C*_*e*_ + *bαR*_1_ > −*bP* and when α*R*_1_ + *M* + *W* − *C*_*e*_ + β(*C*_*R*_ − *P*) + (1 − β)*P* > 0, *E*_*p*_ − *C*_*p*_ + (1 − *r*)*H* + *bηR*_2_ > 0. From Figure 8, it can be seen that the equilibrium point of the game between health information providers and managers of the mHealth platform is (1, 1). It is shown in Figure 8A that even if the proportion of physicians providing high-quality health information strategies is low, the system can also evolve benignly if guided by the reputation mechanism of the mHealth platform. The higher the ratio, the quicker the system will converge to equilibrium. Based on Figures 8A, B, it can be seen that when the physician group has a net benefit, the equilibrium state will eventually be reached regardless of how much effort is devoted to controlling the quality of health information by the mHealth platform.

## 5. Conclusions and suggestions

### 5.1. Conclusions

We have introduced a behavioral game model between health information content providers and managers on mHealth platforms, examined the evolutionary process and ESS of the behaviors of both sides of the game in different situations, and used numerical simulation technology to analyze the quality of health information on mHealth platforms. The present research shows that it is unstable for managers and providers of health information on mHealth platforms to optimize health information quality unilaterally. The mHealth platform actively controls the quality of health information, and the physician community provides high-quality health information as the ideal evolutionary equilibrium of the model. The main factors that influence behavioral decisions on both sides are the rewards obtained by physicians for providing high-quality health information; physicians' reputation benefits and their conversion coefficient; punishments suffered by physicians; the probability of physicians choosing rent-seeking behavior; costs associated with rent-seeking behavior; physicians' investment contribution rate; the reputation benefits of mHealth platforms; the conversion coefficient of their reputation benefit; the probability of exposure and losses from exposure; the and cost and benefit of optimizing the quality of health information both for mHealth platforms and physicians.

### 5.2. Suggestions

The above findings have important implications for the improvement and sustainable development of health information quality on mHealth platforms.

First, it is necessary to focus on reputation mechanism construction to improve the effect of reputation incentives. mHealth platforms controlling the quality of health information and physicians providing quality health information can enhance the reputation of both parties. Further, reputation as a potential resource can bring more traffic to the platform and more patients to the physicians, thus creating more potential revenue. Therefore, mHealth platforms should pay attention to the construction of a reputation mechanism and increase the corresponding reputation incentive mechanism, such as guiding patients to actively give positive comments after getting help from physicians, setting a higher reputation conversion coefficient to ensure that physicians with a good reputation can get more benefits, and improving the effect of reputation incentives.

Second, it is necessary to increase the cost of rent-seeking and reduce the opportunity for rent-seeking. Public medical literacy should be improved, and physicians should be encouraged to monitor each other and expose physicians' rent-seeking behavior. Moreover, the relevant management should increase penalties for mHealth platforms that choose to harbor physicians' rent-seeking behavior to increase the cost of rent-seeking for physicians. Corresponding laws and supervision mechanisms should be established and improved to provide easy-to-operate and responsive supervision channels for the public and physicians to monitor health information on mHealth platforms. Eventually, a physician reputation file should be established. Violations such as rent-seeking should be recorded in the physician's reputation file to curb physicians' rent-seeking behavior and minimize opportunities for rent-seeking.

Third, it is necessary to improve the reward and punishment incentive mechanism and establish an information supervision mechanism. It is difficult to curb the rent-seeking behavior of doctors only through unilateral rewards and punishments because health information managers and providers on the mHealth platform are unable to optimize the quality of health information unilaterally. When considering reputation benefits, patients' affirmation of physicians and the mHealth platform's rewards for physicians are conducive to optimizing health information quality. Patients will ask physicians more paid questions, so the physicians will receive more benefits and will be more motivated. An increase in the punishment of doctors for providing inferior information, an increase in mHealth platform exposure, and losses from exposure are all also conducive to optimizing the quality of health information. A third-party quality watchdog can be introduced to ensure the timely exposure of false and misleading health information. Therefore, the reputation gains of physicians and mHealth platforms should be considered, and effective reward and punishment mechanisms and monitoring mechanisms should be developed.

The following limitations of this study require further in-depth exploration. First, the health information quality on the mHealth platform mainly depends on the providers and managers of health information, but the behavior of users of health information also indirectly influences this. Therefore, future research could consider introducing user behavior for a more comprehensive study. Second, this paper only has examined the sustainability of mHealth platforms from a theoretical and health information quality perspective, without analyzing specific cases. In the future, empirical methods can be considered to further explore this topic and improve its quality.

## Data availability statement

The original contributions presented in the study are included in the article/Supplementary material, further inquiries can be directed to the corresponding author.

## Author contributions

XS: conceptualization, methodology, investigation, validation, software, and writing the original draft. WZ: methodology, formal analysis, writing, reviewing, and editing. YF: writing, reviewing, editing, supervision, and funding acquisition. All authors contributed to the article and approved the submitted version.
