# Supplementary material for: Mobile healthcare platforms' sustainability: The perspective of health information quality
Source: Front Public Health. 2023 Jan 6;10:1059252. doi: 10.3389/fpubh.2022.1059252 (PMC9853185; doi:10.3389/fpubh.2022.1059252)
Supplement: Supplementary file 1 [file Table_1.DOCX]

Supplementary Material

function dy=quality(t,y,W,M,α,R1,Ce,Ep,Cp,H,r,R2,η,b,L,β,CR,P)

dy=zeros(2,1);

dy(1)=y(1)*(1-y(1))*(Ep+η*R2+β*CR+b*L-Cp+((1-r)*H-β*CR-(1-b)*η*R2-b*L)*y(2));

dy(2)=y(2)*(1-y(2))*(y(1)*(β*(CR-P)-(β+b)*P+(1-b)*α*R1+M+P)+W-Ce+b*P+b*α*R1);

**FIGURE 2** Case 1（A)

y0=0.1,x0=0.1,W=1,M=1,α=2,R1=2,Ce=1,Ep=1,Cp=1,H=1,r=0.2,R2=1,η=1,b=0.2,L=2,β=0.2,CR=2,P=4;

[T,y]=ode45(@quality,[0:60],[y0,x0],[],W,M,α,R1,Ce,Ep,Cp,H,r,R2,η,b,L,β,CR,P);

y1=y(:,1);

plot(T,y1,'b*');

xlabel('t');

ylabel('y');

hold on;

y0=0.3,x0=0.1;

[T,y]=ode45(@quality,[0:60],[y0,x0],[],W,M,α,R1,Ce,Ep,Cp,H,r,R2,η,b,L,β,CR,P);

y1=y(:,1);

plot(T,y1,'bO');

xlabel('t');

ylabel('y');

hold on;

y0=0.5,x0=0.1;

[T,y]=ode45(@quality,[0:60],[y0,x0],[],W,M,α,R1,Ce,Ep,Cp,H,r,R2,η,b,L,β,CR,P);

y1=y(:,1);

plot(T,y1,'b^');

xlabel('t');

ylabel('y');

hold on;

y0=0.7,x0=0.1;

[T,y]=ode45(@quality,[0:60],[y0,x0],[],W,M,α,R1,Ce,Ep,Cp,H,r,R2,η,b,L,β,CR,P);

y1=y(:,1);

plot(T,y1,'bx');

xlabel('t');

ylabel('y');

hold on;

y0=0.9,x0=0.1;

[T,y]=ode45(@quality,[0:60],[y0,x0],[],W,M,α,R1,Ce,Ep,Cp,H,r,R2,η,b,L,β,CR,P);

y1=y(:,1);

plot(T,y1,'b+');

xlabel('t');

ylabel('y');

hold on;

title('Schematic diagram of evolution');

legend('y0=0.1','y0=0.3','y0=0.5','y0=0.7','y0=0.9')

axis([0 15 -0.2 1.2])

**FIGURE 2** Case 1（B)

y0=0.1,x0=0.9,W=1,M=1,α=2,R1=2,Ce=1,Ep=1,Cp=1,H=1,r=0.2,R2=1,η=1,b=0.2,L=2,β=0.2,CR=2,P=4;

[T,y]=ode45(@quality,[0:60],[y0,x0],[],W,M,α,R1,Ce,Ep,Cp,H,r,R2,η,b,L,β,CR,P);

y1=y(:,1);

plot(T,y1,'b*');

xlabel('t');

ylabel('y');

hold on;

y0=0.3,x0=0.9;

[T,y]=ode45(@quality,[0:60],[y0,x0],[],W,M,α,R1,Ce,Ep,Cp,H,r,R2,η,b,L,β,CR,P);

y1=y(:,1);

plot(T,y1,'bO');

xlabel('t');

ylabel('y');

hold on;

y0=0.5,x0=0.9;

[T,y]=ode45(@quality,[0:60],[y0,x0],[],W,M,α,R1,Ce,Ep,Cp,H,r,R2,η,b,L,β,CR,P);

y1=y(:,1);

plot(T,y1,'b^');

xlabel('t');

ylabel('y');

hold on;

y0=0.7,x0=0.9;

[T,y]=ode45(@quality,[0:60],[y0,x0],[],W,M,α,R1,Ce,Ep,Cp,H,r,R2,η,b,L,β,CR,P);

y1=y(:,1);

plot(T,y1,'bx');

xlabel('t');

ylabel('y');

hold on;

y0=0.9,x0=0.9;

[T,y]=ode45(@quality,[0:60],[y0,x0],[],W,M,α,R1,Ce,Ep,Cp,H,r,R2,η,b,L,β,CR,P);

y1=y(:,1);

plot(T,y1,'b+');

xlabel('t');

ylabel('y');

hold on;

title('Schematic diagram of evolution');

legend('y0=0.1','y0=0.3','y0=0.5','y0=0.7','y0=0.9')

axis([0 15 -0.2 1.2])

**FIGURE 3** Case 2（A)

y0=0.1,x0=0.1,W=1,M=1,α=2,R1=2,Ce=5,Ep=1,Cp=1,H=1,r=0.2,R2=1,η=1,b=0.2,L=2,β=0.2,CR=2,P=4;

[T,y]=ode45(@quality,[0:60],[y0,x0],[],W,M,α,R1,Ce,Ep,Cp,H,r,R2,η,b,L,β,CR,P);

y1=y(:,1);

plot(T,y1,'b*');

xlabel('t');

ylabel('y');

hold on;

y0=0.3,x0=0.1;

[T,y]=ode45(@quality,[0:60],[y0,x0],[],W,M,α,R1,Ce,Ep,Cp,H,r,R2,η,b,L,β,CR,P);

y1=y(:,1);

plot(T,y1,'bO');

xlabel('t');

ylabel('y');

hold on;

y0=0.5,x0=0.1;

[T,y]=ode45(@quality,[0:60],[y0,x0],[],W,M,α,R1,Ce,Ep,Cp,H,r,R2,η,b,L,β,CR,P);

y1=y(:,1);

plot(T,y1,'b^');

xlabel('t');

ylabel('y');

hold on;

y0=0.7,x0=0.1;

[T,y]=ode45(@quality,[0:60],[y0,x0],[],W,M,α,R1,Ce,Ep,Cp,H,r,R2,η,b,L,β,CR,P);

y1=y(:,1);

plot(T,y1,'bx');

xlabel('t');

ylabel('y');

hold on;

y0=0.9,x0=0.1;

[T,y]=ode45(@quality,[0:60],[y0,x0],[],W,M,α,R1,Ce,Ep,Cp,H,r,R2,η,b,L,β,CR,P);

y1=y(:,1);

plot(T,y1,'b+');

xlabel('t');

ylabel('y');

hold on;

title('Schematic diagram of evolution');

legend('y0=0.1','y0=0.3','y0=0.5','y0=0.7','y0=0.9')

axis([0 15 -0.2 1.2])

**FIGURE 3** Case 2（B)

y0=0.1,x0=0.9,W=1,M=1,α=2,R1=2,Ce=5,Ep=1,Cp=1,H=1,r=0.2,R2=1,η=1,b=0.2,L=2,β=0.2,CR=2,P=4;

[T,y]=ode45(@quality,[0:60],[y0,x0],[],W,M,α,R1,Ce,Ep,Cp,H,r,R2,η,b,L,β,CR,P);

y1=y(:,1);

plot(T,y1,'b*');

xlabel('t');

ylabel('y');

hold on;

y0=0.3,x0=0.9;

[T,y]=ode45(@quality,[0:60],[y0,x0],[],W,M,α,R1,Ce,Ep,Cp,H,r,R2,η,b,L,β,CR,P);

y1=y(:,1);

plot(T,y1,'bO');

xlabel('t');

ylabel('y');

hold on;

y0=0.5,x0=0.9;

[T,y]=ode45(@quality,[0:60],[y0,x0],[],W,M,α,R1,Ce,Ep,Cp,H,r,R2,η,b,L,β,CR,P);

y1=y(:,1);

plot(T,y1,'b^');

xlabel('t');

ylabel('y');

hold on;

y0=0.7,x0=0.9;

[T,y]=ode45(@quality,[0:60],[y0,x0],[],W,M,α,R1,Ce,Ep,Cp,H,r,R2,η,b,L,β,CR,P);

y1=y(:,1);

plot(T,y1,'bx');

xlabel('t');

ylabel('y');

hold on;

y0=0.9,x0=0.9;

[T,y]=ode45(@quality,[0:60],[y0,x0],[],W,M,α,R1,Ce,Ep,Cp,H,r,R2,η,b,L,β,CR,P);

y1=y(:,1);

plot(T,y1,'b+');

xlabel('t');

ylabel('y');

hold on;

title('Schematic diagram of evolution');

legend('y0=0.1','y0=0.3','y0=0.5','y0=0.7','y0=0.9')

axis([0 15 -0.2 1.2])

**FIGURE 4** Case 3（A)

y0=0.1,x0=0.1,W=1,M=1,α=2,R1=2,Ce=5,Ep=1,Cp=3,H=3,r=0.2,R2=1,η=1,b=0.2,L=2,β=0.2,CR=0.1,P=0.04;

[T,y]=ode45(@quality,[0:60],[y0,x0],[],W,M,α,R1,Ce,Ep,Cp,H,r,R2,η,b,L,β,CR,P);

y1=y(:,1);

plot(T,y1,'b*');

xlabel('t');

ylabel('y');

hold on;

y0=0.3,x0=0.1;

[T,y]=ode45(@quality,[0:60],[y0,x0],[],W,M,α,R1,Ce,Ep,Cp,H,r,R2,η,b,L,β,CR,P);

y1=y(:,1);

plot(T,y1,'bO');

xlabel('t');

ylabel('y');

hold on;

y0=0.5,x0=0.1;

[T,y]=ode45(@quality,[0:60],[y0,x0],[],W,M,α,R1,Ce,Ep,Cp,H,r,R2,η,b,L,β,CR,P);

y1=y(:,1);

plot(T,y1,'b^');

xlabel('t');

ylabel('y');

hold on;

y0=0.7,x0=0.1;

[T,y]=ode45(@quality,[0:60],[y0,x0],[],W,M,α,R1,Ce,Ep,Cp,H,r,R2,η,b,L,β,CR,P);

y1=y(:,1);

plot(T,y1,'bx');

xlabel('t');

ylabel('y');

hold on;

y0=0.9,x0=0.1;

[T,y]=ode45(@quality,[0:60],[y0,x0],[],W,M,α,R1,Ce,Ep,Cp,H,r,R2,η,b,L,β,CR,P);

y1=y(:,1);

plot(T,y1,'b+');

xlabel('t');

ylabel('y');

hold on;

title('Schematic diagram of evolution');

legend('y0=0.1','y0=0.3','y0=0.5','y0=0.7','y0=0.9')

axis([0 15 -0.2 1.2])

**FIGURE 4** Case 3（B)

y0=0.1,x0=0.9,W=1,M=1,α=2,R1=2,Ce=5,Ep=1,Cp=3,H=3,r=0.2,R2=1,η=1,b=0.2,L=2,β=0.2,CR=0.1,P=0.04;

[T,y]=ode45(@quality,[0:60],[y0,x0],[],W,M,α,R1,Ce,Ep,Cp,H,r,R2,η,b,L,β,CR,P);

y1=y(:,1);

plot(T,y1,'b*');

xlabel('t');

ylabel('y');

hold on;

y0=0.3,x0=0.9;

[T,y]=ode45(@quality,[0:60],[y0,x0],[],W,M,α,R1,Ce,Ep,Cp,H,r,R2,η,b,L,β,CR,P);

y1=y(:,1);

plot(T,y1,'bO');

xlabel('t');

ylabel('y');

hold on;

y0=0.5,x0=0.9;

[T,y]=ode45(@quality,[0:60],[y0,x0],[],W,M,α,R1,Ce,Ep,Cp,H,r,R2,η,b,L,β,CR,P);

y1=y(:,1);

plot(T,y1,'b^');

xlabel('t');

ylabel('y');

hold on;

y0=0.7,x0=0.9;

[T,y]=ode45(@quality,[0:60],[y0,x0],[],W,M,α,R1,Ce,Ep,Cp,H,r,R2,η,b,L,β,CR,P);

y1=y(:,1);

plot(T,y1,'bx');

xlabel('t');

ylabel('y');

hold on;

y0=0.9,x0=0.9;

[T,y]=ode45(@quality,[0:60],[y0,x0],[],W,M,α,R1,Ce,Ep,Cp,H,r,R2,η,b,L,β,CR,P);

y1=y(:,1);

plot(T,y1,'b+');

xlabel('t');

ylabel('y');

hold on;

title('Schematic diagram of evolution');

legend('y0=0.1','y0=0.3','y0=0.5','y0=0.7','y0=0.9')

axis([0 15 -0.2 1.2])

**FIGURE 4** Case 3（C)

y0=0.1,x0=0.1,W=1,M=1,α=2,R1=9,Ce=5,Ep=1,Cp=3,H=3,r=0.2,R2=1,η=1,b=0.2,L=2,β=0.2,CR=0.1,P=0.04;

[T,y]=ode45(@quality,[0:60],[y0,x0],[],W,M,α,R1,Ce,Ep,Cp,H,r,R2,η,b,L,β,CR,P);

y1=y(:,1);

plot(T,y1,'b*');

xlabel('t');

ylabel('y');

hold on;

y0=0.3,x0=0.1;

[T,y]=ode45(@quality,[0:60],[y0,x0],[],W,M,α,R1,Ce,Ep,Cp,H,r,R2,η,b,L,β,CR,P);

y1=y(:,1);

plot(T,y1,'bO');

xlabel('t');

ylabel('y');

hold on;

y0=0.5,x0=0.1;

[T,y]=ode45(@quality,[0:60],[y0,x0],[],W,M,α,R1,Ce,Ep,Cp,H,r,R2,η,b,L,β,CR,P);

y1=y(:,1);

plot(T,y1,'b^');

xlabel('t');

ylabel('y');

hold on;

y0=0.7,x0=0.1;

[T,y]=ode45(@quality,[0:60],[y0,x0],[],W,M,α,R1,Ce,Ep,Cp,H,r,R2,η,b,L,β,CR,P);

y1=y(:,1);

plot(T,y1,'bx');

xlabel('t');

ylabel('y');

hold on;

y0=0.9,x0=0.1;

[T,y]=ode45(@quality,[0:60],[y0,x0],[],W,M,α,R1,Ce,Ep,Cp,H,r,R2,η,b,L,β,CR,P);

y1=y(:,1);

plot(T,y1,'b+');

xlabel('t');

ylabel('y');

hold on;

title('Schematic diagram of evolution');

legend('y0=0.1','y0=0.3','y0=0.5','y0=0.7','y0=0.9')

axis([0 15 -0.2 1.2])

**FIGURE 4** Case 3（C)

y0=0.1,x0=0.1,W=1,M=1,α=9,R1=2,Ce=5,Ep=1,Cp=3,H=3,r=0.2,R2=1,η=1,b=0.2,L=2,β=0.2,CR=0.1,P=0.04;

[T,y]=ode45(@quality,[0:60],[y0,x0],[],W,M,α,R1,Ce,Ep,Cp,H,r,R2,η,b,L,β,CR,P);

y1=y(:,1);

plot(T,y1,'b*');

xlabel('t');

ylabel('y');

hold on;

y0=0.3,x0=0.1;

[T,y]=ode45(@quality,[0:60],[y0,x0],[],W,M,α,R1,Ce,Ep,Cp,H,r,R2,η,b,L,β,CR,P);

y1=y(:,1);

plot(T,y1,'bO');

xlabel('t');

ylabel('y');

hold on;

y0=0.5,x0=0.1;

[T,y]=ode45(@quality,[0:60],[y0,x0],[],W,M,α,R1,Ce,Ep,Cp,H,r,R2,η,b,L,β,CR,P);

y1=y(:,1);

plot(T,y1,'b^');

xlabel('t');

ylabel('y');

hold on;

y0=0.7,x0=0.1;

[T,y]=ode45(@quality,[0:60],[y0,x0],[],W,M,α,R1,Ce,Ep,Cp,H,r,R2,η,b,L,β,CR,P);

y1=y(:,1);

plot(T,y1,'bx');

xlabel('t');

ylabel('y');

hold on;

y0=0.9,x0=0.1;

[T,y]=ode45(@quality,[0:60],[y0,x0],[],W,M,α,R1,Ce,Ep,Cp,H,r,R2,η,b,L,β,CR,P);

y1=y(:,1);

plot(T,y1,'b+');

xlabel('t');

ylabel('y');

hold on;

title('Schematic diagram of evolution');

legend('y0=0.1','y0=0.3','y0=0.5','y0=0.7','y0=0.9')

axis([0 15 -0.2 1.2])

**FIGURE 4** Case 3（D)

y0=0.1,x0=0.1,W=1,M=1,α=2,R1=2,Ce=5,Ep=1,Cp=3,H=3,r=0.2,R2=1,η=1,b=0.2,L=2,β=0.2,CR=2.9,P=0.04;

[T,y]=ode45(@quality,[0:60],[y0,x0],[],W,M,α,R1,Ce,Ep,Cp,H,r,R2,η,b,L,β,CR,P);

y1=y(:,1);

plot(T,y1,'b*');

xlabel('t');

ylabel('y');

hold on;

y0=0.3,x0=0.1;

[T,y]=ode45(@quality,[0:60],[y0,x0],[],W,M,α,R1,Ce,Ep,Cp,H,r,R2,η,b,L,β,CR,P);

y1=y(:,1);

plot(T,y1,'bO');

xlabel('t');

ylabel('y');

hold on;

y0=0.5,x0=0.1;

[T,y]=ode45(@quality,[0:60],[y0,x0],[],W,M,α,R1,Ce,Ep,Cp,H,r,R2,η,b,L,β,CR,P);

y1=y(:,1);

plot(T,y1,'b^');

xlabel('t');

ylabel('y');

hold on;

y0=0.7,x0=0.1;

[T,y]=ode45(@quality,[0:60],[y0,x0],[],W,M,α,R1,Ce,Ep,Cp,H,r,R2,η,b,L,β,CR,P);

y1=y(:,1);

plot(T,y1,'bx');

xlabel('t');

ylabel('y');

hold on;

y0=0.9,x0=0.1;

[T,y]=ode45(@quality,[0:60],[y0,x0],[],W,M,α,R1,Ce,Ep,Cp,H,r,R2,η,b,L,β,CR,P);

y1=y(:,1);

plot(T,y1,'b+');

xlabel('t');

ylabel('y');

hold on;

title('Schematic diagram of evolution');

legend('y0=0.1','y0=0.3','y0=0.5','y0=0.7','y0=0.9')

axis([0 15 -0.2 1.2])

**FIGURE 4** Case 3（E)

y0=0.1,x0=0.1,W=1,M=1,α=2,R1=2,Ce=5,Ep=1,Cp=3,H=3,r=0.2,R2=1,η=1,b=0.2,L=2,β=0.2,CR=0.1,P=4;

[T,y]=ode45(@quality,[0:60],[y0,x0],[],W,M,α,R1,Ce,Ep,Cp,H,r,R2,η,b,L,β,CR,P);

y1=y(:,1);

plot(T,y1,'b*');

xlabel('t');

ylabel('y');

hold on;

y0=0.3,x0=0.1;

[T,y]=ode45(@quality,[0:60],[y0,x0],[],W,M,α,R1,Ce,Ep,Cp,H,r,R2,η,b,L,β,CR,P);

y1=y(:,1);

plot(T,y1,'bO');

xlabel('t');

ylabel('y');

hold on;

y0=0.5,x0=0.1;

[T,y]=ode45(@quality,[0:60],[y0,x0],[],W,M,α,R1,Ce,Ep,Cp,H,r,R2,η,b,L,β,CR,P);

y1=y(:,1);

plot(T,y1,'b^');

xlabel('t');

ylabel('y');

hold on;

y0=0.7,x0=0.1;

[T,y]=ode45(@quality,[0:60],[y0,x0],[],W,M,α,R1,Ce,Ep,Cp,H,r,R2,η,b,L,β,CR,P);

y1=y(:,1);

plot(T,y1,'bx');

xlabel('t');

ylabel('y');

hold on;

y0=0.9,x0=0.1;

[T,y]=ode45(@quality,[0:60],[y0,x0],[],W,M,α,R1,Ce,Ep,Cp,H,r,R2,η,b,L,β,CR,P);

y1=y(:,1);

plot(T,y1,'b+');

xlabel('t');

ylabel('y');

hold on;

title('Schematic diagram of evolution');

legend('y0=0.1','y0=0.3','y0=0.5','y0=0.7','y0=0.9')

axis([0 15 -0.2 1.2])

**FIGURE 4** Case 3（F)

y0=0.1,x0=0.1,W=1,M=1,α=2,R1=2,Ce=5,Ep=1,Cp=3,H=3,r=0.2,R2=1,η=1,b=0.45,L=2,β=0.2,CR=0.1,P=0.04;

[T,y]=ode45(@quality,[0:60],[y0,x0],[],W,M,α,R1,Ce,Ep,Cp,H,r,R2,η,b,L,β,CR,P);

y1=y(:,1);

plot(T,y1,'b*');

xlabel('t');

ylabel('y');

hold on;

y0=0.3,x0=0.1;

[T,y]=ode45(@quality,[0:60],[y0,x0],[],W,M,α,R1,Ce,Ep,Cp,H,r,R2,η,b,L,β,CR,P);

y1=y(:,1);

plot(T,y1,'bO');

xlabel('t');

ylabel('y');

hold on;

y0=0.5,x0=0.1;

[T,y]=ode45(@quality,[0:60],[y0,x0],[],W,M,α,R1,Ce,Ep,Cp,H,r,R2,η,b,L,β,CR,P);

y1=y(:,1);

plot(T,y1,'b^');

xlabel('t');

ylabel('y');

hold on;

y0=0.7,x0=0.1;

[T,y]=ode45(@quality,[0:60],[y0,x0],[],W,M,α,R1,Ce,Ep,Cp,H,r,R2,η,b,L,β,CR,P);

y1=y(:,1);

plot(T,y1,'bx');

xlabel('t');

ylabel('y');

hold on;

y0=0.9,x0=0.1;

[T,y]=ode45(@quality,[0:60],[y0,x0],[],W,M,α,R1,Ce,Ep,Cp,H,r,R2,η,b,L,β,CR,P);

y1=y(:,1);

plot(T,y1,'b+');

xlabel('t');

ylabel('y');

hold on;

title('Schematic diagram of evolution');

legend('y0=0.1','y0=0.3','y0=0.5','y0=0.7','y0=0.9')

axis([0 15 -0.2 1.2])

**FIGURE 4** Case 3（G)

y0=0.1,x0=0.1,W=1,M=1,α=2,R1=2,Ce=5,Ep=1,Cp=3,H=3,r=0.2,R2=1,η=1,b=0.2,L=4.85,β=0.2,CR=0.1,P=0.04;

[T,y]=ode45(@quality,[0:60],[y0,x0],[],W,M,α,R1,Ce,Ep,Cp,H,r,R2,η,b,L,β,CR,P);

y1=y(:,1);

plot(T,y1,'b*');

xlabel('t');

ylabel('y');

hold on;

y0=0.3,x0=0.1;

[T,y]=ode45(@quality,[0:60],[y0,x0],[],W,M,α,R1,Ce,Ep,Cp,H,r,R2,η,b,L,β,CR,P);

y1=y(:,1);

plot(T,y1,'bO');

xlabel('t');

ylabel('y');

hold on;

y0=0.5,x0=0.1;

[T,y]=ode45(@quality,[0:60],[y0,x0],[],W,M,α,R1,Ce,Ep,Cp,H,r,R2,η,b,L,β,CR,P);

y1=y(:,1);

plot(T,y1,'b^');

xlabel('t');

ylabel('y');

hold on;

y0=0.7,x0=0.1;

[T,y]=ode45(@quality,[0:60],[y0,x0],[],W,M,α,R1,Ce,Ep,Cp,H,r,R2,η,b,L,β,CR,P);

y1=y(:,1);

plot(T,y1,'bx');

xlabel('t');

ylabel('y');

hold on;

y0=0.9,x0=0.1;

[T,y]=ode45(@quality,[0:60],[y0,x0],[],W,M,α,R1,Ce,Ep,Cp,H,r,R2,η,b,L,β,CR,P);

y1=y(:,1);

plot(T,y1,'b+');

xlabel('t');

ylabel('y');

hold on;

title('Schematic diagram of evolution');

legend('y0=0.1','y0=0.3','y0=0.5','y0=0.7','y0=0.9')

axis([0 15 -0.2 1.2])

**FIGURE 5** Case 4（A)

y0=0.1,x0=0.1,W=1,M=1,α=2,R1=2,Ce=1,Ep=1,Cp=3,H=3,r=0.2,R2=1,η=1,b=0.2,L=2,β=0.2,CR=2,P=4;

[T,y]=ode45(@quality,[0:60],[y0,x0],[],W,M,α,R1,Ce,Ep,Cp,H,r,R2,η,b,L,β,CR,P);

y1=y(:,1);

plot(T,y1,'b*');

xlabel('t');

ylabel('y');

hold on;

y0=0.3,x0=0.1;

[T,y]=ode45(@quality,[0:60],[y0,x0],[],W,M,α,R1,Ce,Ep,Cp,H,r,R2,η,b,L,β,CR,P);

y1=y(:,1);

plot(T,y1,'bO');

xlabel('t');

ylabel('y');

hold on;

y0=0.5,x0=0.1;

[T,y]=ode45(@quality,[0:60],[y0,x0],[],W,M,α,R1,Ce,Ep,Cp,H,r,R2,η,b,L,β,CR,P);

y1=y(:,1);

plot(T,y1,'b^');

xlabel('t');

ylabel('y');

hold on;

y0=0.7,x0=0.1;

[T,y]=ode45(@quality,[0:60],[y0,x0],[],W,M,α,R1,Ce,Ep,Cp,H,r,R2,η,b,L,β,CR,P);

y1=y(:,1);

plot(T,y1,'bx');

xlabel('t');

ylabel('y');

hold on;

y0=0.9,x0=0.1;

[T,y]=ode45(@quality,[0:60],[y0,x0],[],W,M,α,R1,Ce,Ep,Cp,H,r,R2,η,b,L,β,CR,P);

y1=y(:,1);

plot(T,y1,'b+');

xlabel('t');

ylabel('y');

hold on;

title('Schematic diagram of evolution');

legend('y0=0.1','y0=0.3','y0=0.5','y0=0.7','y0=0.9')

axis([0 15 -0.2 1.2])

**FIGURE 5** Case 4（B)

y0=0.1,x0=0.9,W=1,M=1,α=2,R1=2,Ce=1,Ep=1,Cp=3,H=3,r=0.2,R2=1,η=1,b=0.2,L=2,β=0.2,CR=2,P=4;

[T,y]=ode45(@quality,[0:60],[y0,x0],[],W,M,α,R1,Ce,Ep,Cp,H,r,R2,η,b,L,β,CR,P);

y1=y(:,1);

plot(T,y1,'b*');

xlabel('t');

ylabel('y');

hold on;

y0=0.3,x0=0.9;

[T,y]=ode45(@quality,[0:60],[y0,x0],[],W,M,α,R1,Ce,Ep,Cp,H,r,R2,η,b,L,β,CR,P);

y1=y(:,1);

plot(T,y1,'bO');

xlabel('t');

ylabel('y');

hold on;

y0=0.5,x0=0.9;

[T,y]=ode45(@quality,[0:60],[y0,x0],[],W,M,α,R1,Ce,Ep,Cp,H,r,R2,η,b,L,β,CR,P);

y1=y(:,1);

plot(T,y1,'b^');

xlabel('t');

ylabel('y');

hold on;

y0=0.7,x0=0.9;

[T,y]=ode45(@quality,[0:60],[y0,x0],[],W,M,α,R1,Ce,Ep,Cp,H,r,R2,η,b,L,β,CR,P);

y1=y(:,1);

plot(T,y1,'bx');

xlabel('t');

ylabel('y');

hold on;

y0=0.9,x0=0.9;

[T,y]=ode45(@quality,[0:60],[y0,x0],[],W,M,α,R1,Ce,Ep,Cp,H,r,R2,η,b,L,β,CR,P);

y1=y(:,1);

plot(T,y1,'b+');

xlabel('t');

ylabel('y');

hold on;

title('Schematic diagram of evolution');

legend('y0=0.1','y0=0.3','y0=0.5','y0=0.7','y0=0.9')

axis([0 15 -0.2 1.2])
